# Strengthening self-care agency in pregnancy: A new approach to improve maternal health outcomes in low-and middle-income countries

**DOI:** 10.3389/fpubh.2022.968375

**Published:** 2022-07-28

**Authors:** Anam Shahil Feroz

**Affiliations:** ^1^Institute of Health Policy Management and Evaluation, University of Toronto, Toronto, ON, Canada; ^2^Community Health Sciences Department, Aga Khan University, Karachi, Pakistan

**Keywords:** self-care, pregnancy, self-care agency, low-middle-income countries (LMIC), maternal health outcomes

Maternal mortality is unacceptably high with the vast majority of these deaths (94%) occurring in low-middle-income countries (1). It is well established that most maternal deaths are preventable, as the health-care solutions to prevent or manage complications are well known (2). Pregnant women in remote areas are the least likely to receive adequate maternal health care services (1). This is especially true for regions such as sub-Saharan Africa and South Asia which have low numbers of skilled health workers (1). World Health Organization is working to contribute to the reduction of maternal mortality by (1) addressing inequalities in access to and quality of maternal health care services; (2) ensuring universal health coverage for comprehensive reproductive, maternal, and newborn health care; (3) addressing all causes of maternal mortality; (4) strengthening health systems to collect high-quality data to respond to the needs and priorities of women and girls; and (5) ensuring accountability to improve quality of care and equity (1). A factor missing in this discourse is an analysis of self-care agency among pregnant women as a determinant of improved maternal health outcomes.

This opinion piece makes a unique contribution to the scientific literature by recommending a new approach to improve maternal health outcomes in low-middle-income countries. This opinion piece intends to highlight the role and significance of the self-care agency in enabling pregnant women to perform prenatal care actions to improve maternal health outcomes. The piece will briefly discuss the linkage between self-care agency and self-care, the reasons for poor self-care agency among pregnant women in low-middle income countries, and suggest pathways through which self-care agency among pregnant women could be strengthened to improve maternal health outcomes in low-middle-income countries. Rather than offering guidelines, this opinion piece intends to launch a much-needed dialogue amongst interested maternal health experts, health service researchers, public health scholars, and clinicians who are experiencing difficulties in improving maternal health outcomes among pregnant women, especially in low-middle-income countries.

We all are seeing an increase in momentum in terms of the use of digital health solutions to enable pregnant women to perform self-care interventions (3–5). Although self-care interventions offer innovative and equitable ways to improve maternal health outcomes (6), the uptake of self-care interventions among pregnant women is low, especially in low-middle-income countries (7). Notably, pregnant women need to have a self-care agency to perform self-care interventions throughout pregnancy (8). Self-care agency, one of the main concepts in Dorothea Orem's general theory of nursing, is defined as the “acquired ability to perform self-care” (9). Self-care agency develops from childhood, reaches maturity in adulthood, and declines with old age (9). Self-care agency is viewed as influencing self-care during pregnancy, and, in turn, self-care is viewed as influencing maternal health care outcomes (8, 10). Thus, poor self-care agency among pregnant women compromises women's ability to perform self-care interventions necessary to attain improved maternal health outcomes.

In low-middle-income countries, various demographic and sociocultural factors, as well as developmental stressors, heavily influence pregnant women's self-care agency (8, 11, 12). First, the demographic factors such as maternal age, parity, gestational age, economic status, educational level, employment status, marital status, and distance to a health facility greatly influence pregnant women's self-care agency and, in turn, the uptake of self-care interventions (8). Hart and Foster's correlational study on self-care agency among pregnant women compared the scores of two different groups of pregnant women (12). One group consisted of clinic patients mainly of lower socioeconomic background with at most a high school education whereas the other group consisted of women who were college-educated and had private insurance. The study found that the self-care agency scores were significantly different between the two groups and that the educational level and type of insurance had an impact on self-care agency scores (12). Second, the complex interaction of sociocultural factors including societal structures, cultural practices, religious beliefs, gendered imbalances in decision making, women's restricted access to resources, and restricted women's mobility impact pregnant women's self-care agency to a considerable extent (8). All these demographic and sociocultural factors compromise a woman's ability to adopt healthy behaviors during pregnancy and perform self-care actions which are critical for ensuring safe pregnancies. For example, Lowe et al. (13) qualitative study revealed that pregnant women in Gambia have unfavorable position within the household and thus have limited control over material and social resources such as freedom to use the horse-cart to obtain healthcare services (13).Third, the developmental stress perceived by pregnant women during pregnancy also impacts the self-care agency of women (11). Pregnancy is a period of transition with important physiological and emotional changes in which women perceive developmental stressors due to increased healthcare needs. Can et al. study on factors affecting self-care agency in pregnancy found that self-care agency decreased as the developmental stress increased over the course of pregnancy (11).

We are at the point where we can make the turn toward a new approach to improve maternal healthcare in low-middle-income countries. The new approach involves strengthening the self-care agency of pregnant women in low-and-middle-income countries to enable women to perform self-care actions during pregnancy. There are some pathways to strengthen the self-care agency of pregnant women such as training clinicians to improve women's ability to practice self-care during pregnancy (11), adopting digital health technologies to empower women to perform self-care at home, and coaching clinicians to identify areas where pregnant women self-care agency is compromised (8, 12). First, there is an opportunity to train clinicians and community health workers to enhance pregnant women's capacity to practice self-care during pregnancy. Clinicians especially nurses can educate and train women during antenatal visits about basic prenatal care actions including obtaining health supervision and education, maintaining nutrition, balancing rest and activity, abstaining from hazards, and maintaining social interactions (11). For instance, Geldsetzer et al. study in Dar es Salaam, Tanzania involved community health workers for counseling pregnant women on maternal health during home visits with the aim to improve self-care and management among pregnant women (14, 15). Second, researchers can capitalize on increasing cell phone penetration among pregnant women given that digital health technologies hold the most promise for empowering and supporting pregnant women thereby improving their self-care agency. For instance, Parsa et al. implemented a mobile application in Iran to improve self-care among preeclamptic women. The study found out that the mobile intervention enabled pregnant women to identify signs and symptoms, resulting in the early detection and management of their condition, and likely reduction of its adverse consequences (16). Third, clinicians can be coached to identify areas where pregnant women's self-care agency is compromised and explore pathways for strengthening their self-care agency (8, 12). For instance, the nurses at the antenatal clinics could speak to pregnant women to learn about their motivation to care for themselves, decision-making skills for seeking maternal healthcare, and awareness of pregnancy, to intervene appropriately. Feroz et al. study explored the needs, requirements, and preferences of telemonitoring use among pregnant women at high risk for preeclampsia in Pakistan (17). Most pregnant women in the study embraced the telemonitoring program and perceived a large opportunity to establish a technology to improve their self-care agency (17).

Given the dominant role of the self-care agency in improving maternal health outcomes. Future research should: (1) conduct a longitudinal study to measure self-care agency over the course of pregnancy in low-middle-income countries using an appraisal of the self-care agency scale; (2) clarify the concept of self-care agency in pregnancy, particularly in relation to low-middle-income country context; and (3) investigate specific demographic, sociocultural, behavioral and developmental factors that influence self-care agency among pregnant women and identify pathways to improve self-care agency during pregnancy.

## Author contributions

AS conceptualized the idea and wrote the manuscript.

## Conflict of interest

The author declares that the research was conducted in the absence of any commercial or financial relationships that could be construed as a potential conflict of interest.

## Publisher's note

All claims expressed in this article are solely those of the authors and do not necessarily represent those of their affiliated organizations, or those of the publisher, the editors and the reviewers. Any product that may be evaluated in this article, or claim that may be made by its manufacturer, is not guaranteed or endorsed by the publisher.
